# Development of an intervention to improve appropriate polypharmacy in older people in primary care using a theory-based method

**DOI:** 10.1186/s12913-016-1907-3

**Published:** 2016-11-16

**Authors:** Cathal A. Cadogan, Cristín Ryan, Jill J. Francis, Gerard J. Gormley, Peter Passmore, Ngaire Kerse, Carmel M. Hughes

**Affiliations:** 1 School of Pharmacy, Royal College of Surgeons in Ireland, Dublin, Ireland; 2 School of Health Sciences, City University London, London, UK; 3 Department of General Practice, Queen’s University Belfast, Belfast, UK; 4 Centre for Public Health, Queen’s University Belfast, Belfast, UK; 5 School of Population Health, University of Auckland, Auckland, New Zealand; 6 School of Pharmacy, Queen’s University Belfast, 97 Lisburn Road, Belfast, BT9 7BL UK

**Keywords:** Polypharmacy, Intervention, Behaviour change, Prescribing, Dispensing, Theoretical domains framework, Feasibility, APEASE

## Abstract

**Background:**

It is advocated that interventions to improve clinical practice should be developed using a systematic approach and intervention development methods should be reported. However, previous interventions aimed at ensuring that older people receive appropriate polypharmacy have lacked details on their development. This study formed part of a multiphase research project which aimed to develop an intervention to improve appropriate polypharmacy in older people in primary care.

**Methods:**

The target behaviours for the intervention were prescribing and dispensing of appropriate polypharmacy to older patients by general practitioners (GPs) and community pharmacists. Intervention development followed a systematic approach, including previous mapping of behaviour change techniques (BCTs) to key domains from the Theoretical Domains Framework that were perceived by GPs and pharmacists to influence the target behaviours. Draft interventions were developed to operationalise selected BCTs through team discussion. Selection of an intervention for feasibility testing was guided by a subset of the APEASE (Affordability, Practicability, Effectiveness/cost-effectiveness, Acceptability, Side-effects/safety, Equity) criteria.

**Results:**

Three draft interventions comprising selected BCTs were developed, targeting patients, pharmacists and GPs, respectively. Following assessment of each intervention using a subset of the APEASE criteria (affordability, practicability, acceptability), the GP-targeted intervention was selected for feasibility testing. This intervention will involve a demonstration of the behaviour and will be delivered as an online video. The video demonstrating how GPs can prescribe appropriate polypharmacy during a typical consultation with an older patient will also demonstrate salience of consequences (feedback emphasising the positive outcomes of performing the behaviour). Action plans and prompts/cues will be used as complementary intervention components. The intervention is designed to facilitate the prescribing of appropriate polypharmacy in routine practice.

**Conclusion:**

A GP-targeted intervention to improve appropriate polypharmacy in older people has been developed using a systematic approach. Intervention content has been specified using an established taxonomy of BCTs and selected to maximise feasibility. The results of a future feasibility study will help to determine if the theory-based intervention requires further refinement before progressing to a larger scale randomised evaluation.

## Background

In developing interventions to improve clinical practice, it is advocated that researchers should adopt a systematic approach and provide explicit reporting of the intervention development process [1]. The UK Medical Research Council’s (MRC) complex intervention framework recommends that intervention development be guided by best available evidence and appropriate theory [2]. Systematic literature reviews can help in identifying, appraising and pooling available evidence. This can aid the selection of intervention components, as well as outcome measures to include as part of the overall evaluation. The use of appropriate theory can help in overcoming inherent limitations where intervention development involves a pragmatic approach based on researchers’ own implicit, and potentially biased, assumptions as to what is likely to be effective [3, 4]. For example, theories can be used to generate testable hypotheses and explore potential causal mechanisms underlying the intervention’s effect. Although established systematic review methodologies exist which have been extensively detailed in the literature, the methods involved in identifying and/or developing appropriate theory are much less clear [5]. A recognised limitation of the MRC framework is that it does not provide guidance on how theory can be incorporated into the intervention development process [6].

Interventions aimed at improving clinical practice often require behaviour change among healthcare professionals (HCPs). For example, a number of studies have focussed on the implementation of different evidence-based guidelines by HCPs as the target behaviour [7–9]. By incorporating behaviour change theory into intervention development, researchers can target causal determinants of behaviour/behaviour change, thus making interventions more likely to be effective [10]. This requires a clear understanding of the target behaviour, as well as knowledge of relevant behaviour change theories, so that specific techniques can then be used as part of the intervention to elicit the required changes. A key challenge faced by researchers from non-health psychology backgrounds involves selecting a theory, or combination of theories, from the vast range of existing psychological theories and using these theories appropriately to understand and change target behaviours.

The development of the Theoretical Domains Framework (TDF) of behaviour change [11] has gone some way to help overcome this challenge. The TDF simplifies psychological theory relevant to behaviour change, in order to make behavioural theories more accessible to researchers from non-health psychology backgrounds [11]. TDF version 1 consists of 12 theoretical domains that are relevant to changing HCPs’ behaviour: ‘Knowledge’; ‘Skills’; ‘Social/professional role and identity’; ‘Beliefs about capabilities’; ‘Beliefs about consequences’; ‘Motivation and goals’; ‘Memory, attention and decision processes’; ‘Environmental context and resources’; ’Social influences’; ‘Emotion’; ‘Behavioural regulation’; ‘Nature of the behaviours’ [11]. Eleven of the twelve theoretical domains are proposed to be mediators of behaviour change with ‘Nature of the Behaviours’ being the exceptional domain which relates to the key characteristics of the behaviour of interest as opposed to potential mediating mechanisms or influences [11, 12]. The TDF has since been refined and version 2 consists of 14 theoretical domains [13]. However, TDF version 1 (12 domains) is still in use [7, 14, 15]. The TDF has been used as part of a systematic approach to intervention development in order to identify key theoretical domains that are perceived to influence HCPs’ behaviours [1]. It provides a theoretically-robust evidence base to inform intervention design whereby domains are mapped to behaviour change techniques (BCTs) which form the intervention’s so-called ‘active ingredients’ [10, 16]. This can help researchers to incorporate a theory-base into the intervention development phase.

The aim of the current study which formed part of a multiphase research project was to develop an intervention to improve appropriate polypharmacy in older people in primary care, drawing on relevant methodological advances in intervention development research, as outlined above. The use of multiple medicines, also termed polypharmacy, is increasingly common in older people [17, 18] and ensuring ‘appropriate polypharmacy’ in this patient cohort, whereby prescribing is evidence-based and reflects patients’ clinical needs, is a challenge faced by practitioners that is of considerable clinical and economic importance, particularly in light of continuing growth in the size of the older population [19]. Polypharmacy has been identified as the principal determinant of potentially inappropriate prescribing (PIP) in older populations [4, 5] and linked to negative clinical consequences, including medication non-adherence, drug-interactions and adverse drug events (ADEs) [6]. PIP in older people also places a considerable financial burden on health services [4, 5]. The challenge of improving appropriate polypharmacy is further compounded by a lack of available evidence and guidelines to inform clinical practice when prescribing for older people who often suffer from more than one chronic condition (*i.e.* multimorbidity) [20].

The approach to intervention development that underpinned this research project aligns with the MRC framework by drawing on evidence and theory. The evidence base is drawn from the findings of an updated Cochrane review of interventions to improve appropriate polypharmacy in older people [21] which identified a limited range of intervention types. These interventions were most commonly pharmaceutical care-based and typically involved HCPs conducting medication reviews. However, the quality of the available evidence was considered low, owing to risks of bias in the included studies, and details of intervention development and delivery were lacking in published reports [21]. Accordingly, a more systematic approach, incorporating both evidence and theory, was recommended for the development of future interventions [21]. The theory base for the current project was drawn from qualitative TDF-based interviews of general practitioners (GPs) and community pharmacists which we have reported elsewhere [22]. During the qualitative interview phase, key theoretical domains were identified and mapped to BCTs that could be used as the basis of a theory- and evidence-based intervention. The current paper builds on this earlier work and outlines the systematic process that we used to develop an intervention using previously selected BCTs.

## Methods

**Fig. 1:**
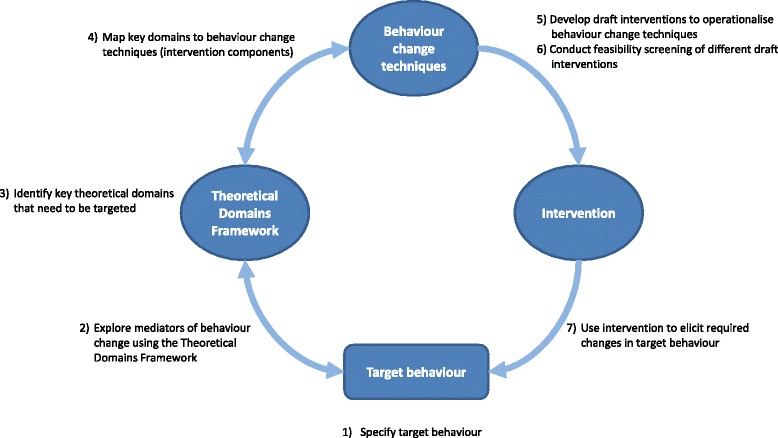
Systematic process of theory-based intervention development

The systematic process of intervention development involved a series of steps, as illustrated in Fig. 1, and was guided by previous related research [1, 23]. The initial steps that laid the foundation for intervention development (*i.e.* TDF-based analysis of target behaviours, mapping of key theoretical domains to BCTs) are reported in detail in a related paper (see Cadogan et al. [22]) and summarised briefly below. The current paper focuses on how selected BCTs were used to develop an intervention that will be tested as part of a future feasibility study. Ethical approval was granted by the Office of Research Ethics Committees Northern Ireland (REC reference 13/NI/0114).

### Initial steps underpinning intervention development process

The prescribing and dispensing of medications are complex processes involving both clinical and non-clinical behaviours [24, 25] and in this project, we focussed on the clinical behaviours performed by GPs and pharmacists within the clinical context of prescribing/dispensing appropriate polypharmacy to older patients (*e.g.* assessing appropriateness of patients’ prescriptions, providing appropriate advice/counselling). Based on the analysis of qualitative TDF-based interviews that were conducted with a purposive sample of HCPs (15 GPs, 15 community pharmacists), eight key domains were selected to be targeted as part of an intervention involving GPs and/or community pharmacists: ‘Skills’, ‘Beliefs about capabilities’, ‘Beliefs about consequences’, ‘Environmental context and resources’, ‘Memory, attention and decision processes’, ‘Social/professional role and identity’, ‘Social influences’, ‘Behavioural regulation’ (see Cadogan et al. [22] for detailed results). The key theoretical domains were selected by the multidisciplinary research team using a consensus-based approach and guided by identified barriers and facilitators within each of the domains that could realistically be targeted using available project resources.

Using previous methodological work whereby experts in the field used consensus-based processes to develop a mapping framework of BCTs that could be used to target specific theoretical domains [10, 16], the research team selected four BCTs for inclusion in an intervention involving GPs and/or community pharmacists to improve appropriate polypharmacy in older people: ‘Action planning’, ‘Prompts/cues’, ‘Modelling or demonstrating of behaviour’, ‘Salience of consequences’. ‘Social support or encouragement’ was selected as an additional BCT for inclusion in a possible community pharmacy-based intervention. The BCT selection process was guided by the interview data (see Cadogan et al. [22] for full details of the mapping process).

### Operationalising behaviour change techniques

Owing to the many ways in which an intervention could be developed using selected BCTs, a discussion exercise was undertaken by the research team to identify the full range of options for operationalising these BCTs (*i.e.* deciding how selected BCTs would be put into practice) [26]. Draft interventions were developed that aimed to ensure the prescribing/dispensing of appropriate polypharmacy to older patients. GPs, community pharmacists and patients were considered as potential intervention targets and/or mediators. There were three important factors that influenced this draft intervention development process, namely, context, evidence and experience, as outlined below.

Firstly, in accordance with available guidance on TDF-based intervention development [1], we considered the local context (particularly current clinical practice), as well as what was likely to be feasible and acceptable to the target group. For example, based on the contextual information from the analysis of the qualitative interviews, it was evident that in order for the intervention to be feasible and acceptable to HCPs, it would have to be time-efficient and easy to incorporate into routine clinical practice. Equally, it was unlikely that a computer-based prescribing alert intervention would be worth pursuing unless issues with the clinical relevance of existing alert systems that were raised during interviews could be addressed [22]. Furthermore, this would have been beyond the scope of the current project’s resources. Secondly, we consulted the updated Cochrane review [21] to determine if evidence existed to inform decisions regarding mode of BCT delivery. Finally, we drew on the multidisciplinary research team’s experience in health psychology, pharmacy and medicine, as well as knowledge of relevant literature to inform decisions about mode of BCT delivery. Based on these considerations, a shortlist of draft interventions was formulated. The shortlist included an outline of each draft intervention’s content and mode of delivery.

### Feasibility screening of draft interventions

Feasibility screening of the draft interventions was undertaken by the research team to select one intervention for further feasibility testing. This process was guided by the APEASE criteria (Affordability, Practicability, Effectiveness/cost-effectiveness, Acceptability, Side-effects/safety, Equity) which have been developed to assist researchers in designing and evaluating interventions [6]. In applying these criteria, we focussed on the subset of criteria that we deemed most relevant to the initial stages of intervention development (i.e. acceptability, practicability and affordability), as opposed to those which we felt would be more applicable to the later stage of intervention evaluation when a full-scale trial is undertaken (i.e. effectiveness, side-effects/safety, equity).

## Results

### Draft intervention development

Based on the team discussion exercise, three draft interventions that operationalised selected BCTs were developed for potential feasibility testing (Table 1). The draft interventions targeted patients, pharmacists and GPs, respectively and focused on increasing the prescribing/dispensing of appropriate polypharmacy to older patients. In developing each draft intervention, we considered the local context (as identified from the qualitative interviews [22]) and attempted to ensure that each intervention would be time-efficient and relatively easy to incorporate into routine clinical practice. We then consulted the updated Cochrane review [21] to determine if evidence existed to inform our decisions regarding mode of BCT delivery. However, as few interventions have previously been trialled in primary care settings and specific intervention components have not been described using standardised terminology, such as BCTs, it was not possible to use the review findings to guide our decision process. Hence, the rationale underpinning our chosen mode of BCT delivery for each intervention was guided by the research team’s experience and knowledge of relevant literature, as outlined below.

**Table 1:** Description of draft interventions

Intervention 1: Patient-targeted intervention
Target group: older patients (≥65 years) receiving polypharmacy
Target behaviour: not applicable^a^
Intervention description: The intervention would be delivered to target patients either through the form of a letter from the GP inviting patients to attend a review consultation (‘Prompts/cues’) or as a coloured label that community pharmacists would attach to patients’ dispensed medication prompting patients to visit their GP for a review consultation (‘Prompts/cues’). GPs would then plan to ensure that patients are prescribed appropriate polypharmacy when they present at the practice (‘Action planning’).
Intervention 2: General practice-based intervention
Target group: GPs
Target behaviour: prescribing of appropriate polypharmacy
Intervention description: The intervention would be delivered through a short online video (or series of videos) demonstrating how GPs can prescribe appropriate polypharmacy during a typical consultation with an older patient (‘Modelling or demonstrating of behaviour’). Each video would last the duration of an average GP consultation (approximately 10 min) and also include feedback from both the GP and patient emphasising the positive outcomes of the consultation (‘Salience of consequences’). As complementary intervention components, GPs would make an explicit plan at practice meetings of when and how they would ensure that target patients are prescribed appropriate polypharmacy (‘Action planning’) and they would be prompted to carry out this plan by the receptionist when target patients present at the practice (‘Prompts/cues’).
Intervention 3: Community pharmacy-based intervention
Target group: community pharmacists
Target behaviour: dispensing of appropriate polypharmacy
Intervention description: The intervention would be delivered through a short online video (or series of videos), similar to that outlined in the GP-based intervention. The video would operationalise two BCTs (‘Modelling or demonstrating of behaviour’, ‘Salience of consequences’) by demonstrating how pharmacists can dispense appropriate polypharmacy during a typical encounter with an older patient and including feedback from both the pharmacist and patient emphasising the positive outcomes of this process.
Patients would be targeted using a collaborative approach between the GP practice and pharmacy. Patients would be identified initially by the GP practice. A list of GP-approved patients would be provided to the pharmacy which would authorise pharmacists to engage with target patients when they present at the pharmacy (‘Social support or encouragement’).
Having been provided with access to the online video(s) together with the list of target patients, pharmacists would make an explicit plan of when and how they would ensure that patients meeting inclusion criteria are dispensed appropriate polypharmacy (‘Action planning’). Pharmacists would be prompted to enact this plan when patients present at the pharmacy either by support staff or a note on the individual patient’s dispensing record (‘Prompts/cues’). Any recommended changes to patients’ current medications would be communicated to the GPs by the pharmacists.

^a^Not applicable: the target behaviour of this draft intervention was not one of the two pre-specified target behaviours (i.e. prescribing and dispensing of appropriate polypharmacy)

#### Patient-targeted intervention

The use of written letters from GPs to deliver the BCT ‘Prompt/cues’ as part of the patient-targeted intervention was based on our knowledge of previous research whereby this mode of intervention delivery has been used to prompt patients to reduce potentially inappropriate long-term benzodiazepine use (*i.e.* a contributing factor to inappropriate polypharmacy in older people) [27].

#### HCP-targeted interventions

The decision to use an online video to deliver the BCT ‘Modelling or demonstrating of behaviour’ as part of both HCP-based interventions (*i.e.* GP and community pharmacist) was informed by a recent project [28]. As part of the IDEA trial (which focused on improving diabetes care through examining, advising and prescribing), a complex intervention that included a video demonstration was developed to target a range of HCP behaviours to improve the quality of care provided to a specific patient population in whom polypharmacy is also common (*i.e.* patients with diabetes mellitus) [28].

The use of the GP-approved list of potential target patients (Table 1) to deliver the BCT ‘Social support or encouragement’ as part of the community pharmacy-based intervention was based on our knowledge of the findings from a previous systematic review in which GP-referral of patients to pharmacists for medication reviews was considered a key element reflecting collaborative care between the two groups of HCPs [29]. Interventions involving more extensive collaboration between GPs and pharmacists are associated with significantly higher implementation rates for recommendations arising from reviews of patients’ medications compared to interventions involving less collaboration [29].

### Feasibility screening assessment

The results of the feasibility screening assessment using the APEASE criteria [6] are shown in Table 2. The patient-targeted intervention was not considered for further evaluation for two main reasons. Firstly, the intervention was intended to target patients’ behaviours as opposed to the pre-specified target behaviours (i.e. prescribing and dispensing of appropriate polypharmacy). Secondly, the intervention was not considered to be a viable option because of issues with its likely acceptability and effectiveness. For example, owing to ethical considerations involved in placing responsibility on patients to seek an appointment with their GP, it was unlikely that the intervention would be deemed acceptable to HCPs. It was also noted that selected BCTs were not specifically targeting the pre-specified target behaviours (*i.e.* prescribing and dispensing of appropriate polypharmacy) as one of the BCTs (*i.e.* ‘Prompt/cue’) was being used to target patients.

**Table 2:** Feasibility screening assessment of draft interventions (guided by a subset of APEASE criteria^a^)

Intervention 1: Patient-targeted intervention
*Strength*: Practicable and affordable intervention. *Limitation*: Intervention does not target the pre-specified target behaviours directly (i.e. prescribing and dispensing of appropriate polypharmacy) and does not operationalise all selected BCTs (*e.g.* ‘Modelling or demonstrating of behaviour’, ‘Salience of consequences’). *Limitation*: Use of ‘Prompts/cues’ as a BCT is not directly targeting HCPs’ behaviour in this context. *Limitation*: Acceptability issues which are likely to limit the potential effectiveness of the intervention (*e.g.* if pharmacists advise patients to make an appointment when GPs do not feel that there are any prescribing issues that need to be addressed).
Intervention 2: General practice-based intervention
*Strength*: Likely to be a practicable and acceptable intervention. *Strength*: Intervention can include all selected BCTs and target HCPs’ prescribing behaviour. *Limitation*: Potential affordability issues with video production costs. *Limitation*: Due to heterogeneity among older patients in terms of comorbidities and prescribed medications, more than one video may be required if intervention is to be effective.
Intervention 3: Community pharmacy-based intervention
*Strength*: Likely to be an acceptable intervention as it acknowledges professional role/boundary related issues between the two groups of HCPs. * Strength*: Intervention can include all selected BCTs. *Limitation*: Potential affordability issues with video production costs. *Limitation*: Intervention is reliant on co-ordination of care between the GP practice and community pharmacy which may impact on practicability and effectiveness. *Limitation*: Due to heterogeneity among older patients in terms of comorbidities and prescribed medications, more than one video may be required if intervention is to be effective.

^a^The subset of the APEASE criteria that were applied during the feasibility screening assessment consisted of affordability, practicability and acceptability

Due to commonalities in the GP and community pharmacist-targeted interventions (*e.g.* online video demonstration), assessments relating to acceptability and affordability were similar. The GP-targeted intervention was selected for further feasibility testing because it was deemed to be more practicable than the pharmacist-led intervention. This was because the pharmacist-led intervention was heavily reliant on the co-ordination of care between general practice and community pharmacy. Thus, if a partnership could not be established between the two healthcare settings, the intervention could not be delivered as intended.

## Discussion

This paper describes the systematic development of an intervention to improve appropriate polypharmacy in older people in primary care and serves to address the lack of theory-based and adequately described interventions in the related literature [21]. The detailed analysis of the target behaviours (i.e. prescribing and dispensing of appropriate polypharmacy) that was undertaken using the TDF [11] as the underpinning theoretical framework enabled us to identify key mediators (*i.e.* barriers, facilitators) of behaviour change to target as part of the intervention [22]. It has been proposed that by targeting specific behaviour change mediators, researchers will enhance the likely effectiveness of interventions [10]. Hence, the intervention was specifically developed to target identified mediators of behaviour change using behaviour change techniques (BCTs) from an established taxonomy [30]. The selection of BCTs was guided by previous methodological work by experts in the field whereby BCTs have been mapped to relevant theoretical domains from the TDF [10, 16] (see Cadogan *et al*. [22] for full details).

In comparison to the initial baseline work (*i.e.* TDF-based analysis of target behaviours, mapping of key theoretical domains to BCTs) for which we were able to draw on established methods and guidance [22], the development of draft interventions using selected BCTs proved to be a challenging process for several reasons. Although the current BCT Taxonomy (version 1) [30] provides definitions of each BCT and illustrative examples of how they can be operationalised, there is no single best approach to decide on how to draft interventions based on a given number of BCTs. A previous review of the TDF-based literature showed that few published studies have progressed through the entire process of TDF-based intervention development [12]. Within the existing literature, the methods used by individual research groups to develop TDF-based interventions following mapping of BCTs to key theoretical domains have varied. For example, French *et al.* [1] reported that, following the mapping process, intervention development was based on a combination of the research team’s experience and feedback from colleagues, while Kolehmainen and Francis [26] reported that an advisory team was established to help inform the process. Hence, it would appear that there is no consensus as to what is the most appropriate procedure for determining how best to operationalise and deliver selected BCTs as part of an intervention.

Ideally, these decisions should be guided by contextual information gathered from key stakeholders (*i.e.* those delivering/receiving intervention) and informed by available evidence. The qualitative HCP interviews helped to provide the former, but despite having updated a Cochrane review [21] as part of the overall project, the findings were of limited value in informing our decision process. Few of the interventions that were included in the review had been conducted specifically in primary care settings and specific intervention components had not been characterised using standardised terminology, such as BCTs. Hence, there was a lack of available evidence on which to base decisions as to how to operationalise and deliver BCTs.

The BCT Taxonomy [30] has been used to retrospectively code interventions identified through systematic reviews [31], in addition to developing behaviour change interventions. Although this will ultimately help to develop an evidence base to inform decisions regarding BCT operationalisation and delivery, this work is still at an early stage. The full potential of characterising behaviour change intervention components in terms of BCTs will not be realised until problems with the reporting of complex interventions are addressed, such as the lack of detailed description of intervention development and delivery that was identified in the Cochrane review [21]. Recent guidance on the reporting of complex interventions [e.g. TIDieR (Template for intervention description and replication) [32], WIDER (Workgroup for Intervention Development and Evaluation Research) [33]] may go some way to help overcome these issues. In the interim, decisions as to how selected BCTs should be operationalised and delivered will rely on the judgement of individual research teams. This process should be based on the team members’ research experience and knowledge of relevant literature while following existing guidance [1] and considering the local context, as well as what is likely to be feasible and acceptable to the target group.

The video demonstration component of the intervention aligned with a ‘work smarter, not harder’ approach that sought to limit any additional workload for GPs in prescribing appropriate polypharmacy [34]. Thus, rather than introducing new behaviours or tasks for GPs to perform, we sought to enable GPs to use available time more efficiently by demonstrating how appropriate polypharmacy can be prescribed during routine consultations with older patients (‘Modelling or demonstrating of behaviour’) and emphasising the potentially positive consequences of performing this behaviour (‘Salience of consequences’). It was envisaged that the video would last the duration of an average GP consultation (i.e. ten minutes) in order to ensure that it was considered clinically authentic by the intervention targets (i.e. GPs). The online mode of delivery was chosen so that GPs could access the video at a time that would be convenient for them. The additional intervention components (*i.e.* action plans, prompts/cues) sought to complement the video demonstration by enabling GPs to overcome the time barriers posed by the existing work environment to performing the target behaviour through the use of action plans and prompts/cues (Table 1). It has been proposed that a ‘work smarter, not harder’ type of approach could enhance the likelihood of achieving improvements in patient care [34].

It must be noted that achieving appropriate polypharmacy in older people is a highly complex clinical challenge and is not limited to changing GPs’ prescribing behaviour alone. A range of other issues need to be addressed in order to optimise medication use in older people, including the lack of available evidence and guidelines to inform clinical practice when prescribing for older multimorbid patients [20] and patient-level barriers when attempting to implement prescribing changes (e.g. disagreement with the appropriateness of prescribing changes) [35]. In light of this complexity, it is unlikely that a single intervention could ever address all of the encompassing issues and challenges. Therefore, a combination of interventions will likely be required. The intention of the intervention developed in the current study was to introduce small, but potentially sustainable, changes in GPs’ current clinical practice aimed at improving prescribing for older people. This is in accordance with available guidance on the development of behaviour change interventions which recommends that change should be introduced incrementally and proposes that building on small successes over time can be more effective than trying to do too much too quickly [6]. If the current intervention proves effective in a future trial evaluation, it could be combined with other interventions to form part of a large multi-strand approach to optimise medication use in older people.

The feasibility screening process was an important methodological step in the development of our intervention as there were several ways in which the combination of BCTs could have been operationalised. The APEASE criteria [6] helped us to consider key factors such as the likely acceptability and practicability of each draft intervention before undertaking any formal feasibility study. For example, due to the anticipated difficulties in the co-ordination of care between general practices and community pharmacies and the negative impact that this would have on the practicability of a community pharmacy-based intervention, the GP-targeted intervention was selected as the most viable option for further evaluation. Applying these criteria also helped us to identify that one of the draft interventions (i.e. the patient-targeted intervention) did not target the pre-specified behaviours that we had set out to change (i.e. prescribing and dispensing of appropriate polypharmacy). A future feasibility study will help us to determine if further refinements to the intervention are required before progressing to a larger scale evaluation in a randomised study.

### Strengths and limitations

The main strength of this study was that it sought to overcome limitations with previously evaluated interventions to improve appropriate polypharmacy in older people [21] by adopting a systematic approach using both evidence and an underlying theory-base as advocated by the MRC framework [2]. Interventions aimed at improving healthcare practice have often not included an underlying theory base [36, 37]. This prevents researchers and clinicians from understanding the mechanisms of change underlying the interventions’ effects. In addition, problems have been noted with the reporting of behaviour change interventions in the literature, such as a lack of detailed descriptions of interventions and the use of inconsistent terminology to characterise intervention content [38]. This makes it difficult to replicate interventions and to either compare the effects of different interventions or to pool data for specific intervention components across studies. The selection of BCTs from an established taxonomy in the current intervention will ensure that the intervention content is described in detail using standardised terminology and this will ultimately help to overcome the above noted limitations of previous research.

As a limitation of our approach, it must be noted that the intervention development work was underpinned by TDF-based qualitative interviews of healthcare professionals which limits the generalisability of the findings [22]. However, participants were sampled across the five administrative health areas in Northern Ireland, and this geographical spread enhanced the transferability of the findings. Given the similarities in general practice across the UK, it is likely that many of the identified barriers and facilitators under each of the theoretical domains would be applicable to GPs across the UK. This claim is supported by the findings of a previous study that compared qualitative TDF-based interview findings of healthcare professionals from different countries, and identified considerable overlap in terms of identified barriers and facilitators under a number of theoretical domains [39].

It must also be noted that there is a degree of subjectivity with the employed intervention development process as selected BCTs could have been operationalised in a number of different ways [1]. Hence, it is uncertain whether another research team would have operationalised selected BCTs in similar ways and produced exact replicates of the interventions that were developed [26]. We attempted to overcome this by drafting a range of interventions through which selected BCTs could be operationalised and selecting an intervention for further testing through the feasibility screening process that was guided by the APEASE criteria [6].

Finally, as the BCT Taxonomy [30] is a relatively new methodological tool, further evidence is required to ensure that particular BCTs achieve behaviour change through the proposed theoretical domains to which they have been mapped by experts in the field [16]. In addition, while our selected BCTs targeted multiple theoretical domains (*e.g.* Modelling or demonstrating the behaviour targeted ‘Skills’, ‘Beliefs about capabilities and ‘Social influences’), it is not yet clear whether targeting an increased number of domains results in more effective interventions [40]. However, by adopting a systematic and theory-based approach to intervention development using the TDF and specifying the intervention’s content in terms of BCTs, we can now make explicit assumptions about the hypothesised mechanisms of change underlying the intervention’s effect [1]. Thus, we can now hypothesise that our GP-targeted intervention that comprises four BCTs will effect change in GPs’ prescribing behaviour to improve appropriate polypharmacy by targeting mediators of behaviour change across eight theoretical domains. As further progress is made in conducting trial evaluations of TDF-based behaviour change interventions, an evidence base will begin to emerge that will help to inform the selection of BCTs to target specific theoretical domains.

## Conclusion

This paper builds on previous work in which we have identified key theoretical domains that were perceived to influence HCPs in prescribing and dispensing appropriate polypharmacy to older people, and mapped these domains to BCTs to include as part of an intervention. Selected BCTs have been operationalised and different draft interventions have been developed. Based on feasibility screening of the draft interventions using the APEASE criteria, a GP-targeted intervention has been selected as the most suitable intervention for further feasibility testing. The results of a feasibility study (ISRCTN18176245) which are currently being evaluated will help to determine if further refinement to the theory-based intervention is required before progressing to a larger scale trial evaluation using a randomised design.

## Abbreviations

ADE: Adverse drug event
APEASE: Affordability Practicability, Effectiveness/cost-effectiveness, Acceptability, Side-effects/safety, Equity
BCT: Behaviour change technique
GP: General practitioner
HCP: Healthcare professional
MRC: Medical Research Council
PIP: Potentially inappropriate prescribing
TDF: Theoretical Domains Framework
TIDieR: Template for intervention description and replication
WIDER: Workgroup for Intervention Development and Evaluation Research
